# Keystone Medical Veterinary Association

**Published:** 1891-02

**Authors:** W. S. Kooker

**Affiliations:** Secretary, Pro. Tem.


*Keystone Veterinary Medical Association.*—The Keystone Veterinary Medical Association held its meeting at College of Physicians, January 3, 1891, President, Dr. Hoskins in the chair. Drs. Zuill, Goentner, Glass, Huidekoper, Weber, Kooker, Eves, Harger, Ridge, Lusson, R. Formad and Drake answered roll call. Minutes of previous meeting were read and approved. The Board of Trustees reported: "In the case of Dr. Huidekoper we beg leave to report as follows: That he issued his statement to his patrons as he found them on his ledger, to veterinary surgeons of his acquaintance, to livery stable keepers who should know of his change of office days and hours and to personal friends. We consider that his having been chosen to a chair in the American Veterinary College and being compelled to be in New York, three days in a week, he was justified in making the announcement. As to the clause "Examination for soundness and diseases of dogs a speciality," we are of the opinion that Dr. Huidekoper has violated no section of the code of ethics, in view of the fact that he issued this as a communication to his patrons and not as an advertisement."

On the question of legality of a member of this association holding a position on the staff of the Veterinary Department of the University of Pennsylvania.

We report that the question of legality, is not one for us to decide; but we disapprove of a member of this association serving on the Board of Managers. The fact of any member continuing in the position shows that he acquiesces in the free treatment advertised by this institution; the principle of which we consider wrong and deserving of censure.

Signed

S. E. Weber, L. O. Lusson,
W. S. Kooker, H. R. Eves.

The trustees reported favorably for membership Drs. A. F. Sellers and A. F. Schreiber. The report received, and the part adopted relative to Drs. Sellers and Schreiber becoming members. Dr. Zuill wished to know how the board of trustees arrived at their conclusion in regard to report as to Dr. Huidekoper's violation of code of ethics. Dr. Kooker read the code of ethics and explained the fact that there was no violation of the code of ethics under the circumstances. Motion being made and seconded that the report, of the trustees, as to Dr. Huidekoper be adopted, it was carried. As to charge against the Clinical Staff of the University, remarks by Dr. Zuill in defense of the staff and by Drs. Sellers, Glass and Hoskins on the question of adopting the report of the trustees were made. Dr. Ridge arguing the point, that the members on the board of managers could not be seperated in the charge from the Clinical Staff. On motion that part of the report of the board of trustees was adopted. On motion the report, as a whole, was adopted.

The following amendment to the Bylaws of the Keystone Medical Association was read and laid over to next meeting. "That any charge as to violation of the Code of Ethics or other charges of a personal nature, against any member of the association shall only be presented in writing, addressed to the secretary of the association, who shall submit the same to the officers and board of trustees of the association, and they shall only be brought before a general meeting of the association and the public, if the officers and board of trustees decide the charge merits further consideration and upon their presentation of the case."

Communication was read from Dr. Webster stating his misfortune and his inability to attend the meetings. Dr. Ridge, a member of the clinical staff presented the following, "moved that the members of the Clinical Staff of the Veterinary Hospital University of Pennsylvania be expelled from this association." Referred to trustees for action.

The bill for three month's rent of room, fifteen dollars was presented, and an order drawn for the amount.

Dr. Sellers read a paper on Pneumonia. In his experience it is a disease attended with little or no danger. Dr. Zuill also agreed with the writer that there was no fatality and very little medication needed. Dr. Kooker recited a case of tetanus when the only cause could be attributed to the dropping of snow water upon the lumber region, followed in a few hours by tetanus in an aggravated form resulting in death in twenty-four hours. Dr.

Hoskins reported a similar case from the same cause. Cases recited by Drs. Eves and Ridge. Adjourned.

W. S. KOOKER, *Secretary*, Pro. Tem.

---

### *Alumni Association of the American Veterinary College.*

### EXECUTIVE COMMITTEE FOR 1891.

| | RESIDENCE. | CLASS. |
|---|---|---|
| Dr. Geo. Bridges (Chairman) | South Norwalk, Ct. | '87 |
| Dr. W. A. Engeman, | 123 St. John's Place, Brooklyn, N. Y. | '88 |
| Dr. W. B. E. Miller, | 532 Penn St., Camden, N. J. | '89 |
| Dr. Jos. Ogle, | 302 West 46th St, New York city, N. Y. | '90 |
| Dr. W. L. Labaw, | 141 West 54th St., New York city, N. Y. | '90 |
| Dr. M. W. Drake, | 312 Reed St., Philadelphia, Pa. | '88 |
| Dr. W. H. Lowe, | Paterson, N. J. | '88 |

### RESIDENT STATE SECRETARIES FOR 1891.

| RESIDENCE. | CLASS. |
|---|---|
| California, W. B. Rowland, D. V. S., Pasadena. | '82 |
| Connecticut, A. A. Tuttle, D. V. S., New Britton. | '85 |
| Colorado, A. F. Martin, D. V. S., Denver. | '81 |
| Delaware, H. B. McDowell, D. V. S., Middletown. | '88 |
| Dakota, G. A. Agersborg, D. V. S., Vermilion. | '81 |
| District of Columbia, Alex. McKenzie, D. V. S., Washington. | '86 |
| Florida, E. A. Child, D. V. S., Leesling. | '81 |
| Iona, A. B. Morse, D. V. S., Des Moines. | '83 |
| Indiana, J. J. Shoemaker, D. V. S., Bluffton. | '87 |
| Illinois, J. W. Harmord, D. V. S., Bloomington. | '88 |
| Indian Territory, G. E. Griffin, D. V. S , Fort Reno. | '89 |
| Kansas, D. C. Ayer, D. V. S., 710 Chenter St., Leavenworth. | '89 |
| Kentucky, J. C. Kidd, D. V. S., Lexington. | '87 |
| Massachusetts, J. J. Winchester, D. V. S., Lawrence. | '78 |
| Maryland, Wm. Dougherty, D. V. S., 1035 Cathedral St., Baltimore. | '76 |
| Maine, G. H. Bailey, D. V. S., 1 Pine St , Portland. | '80 |
| Missouri, T. J. Turner, D. V. S., Mexico. | '87 |
| Michigan, W. J. Menhennitt, D. V. S., Champion. | '90 |
| New Hampshire, A. L. Dodge, D. V. S., Manchester. | '85 |
| New Jersey, J. A. Autenreith, D. V. S., 780 W. Newark ave., J. C. | '82 |
| New York, R. E. Buckley, D. V. S., 210 W. 54th St., New York city. | '87 |
| Ohio, W. M. Tritschler, D. V. S., Dayton, | '88 |
| Oklahoma Territory, C. D. MacMurdo, D. V. S. | '89 |
| Pennsylvania, C. T. Goentner, D. V. S., Beyre Manor. | '81 |
| Rhode Island, W. L. Brut, D. V. S., Providence. | '81 |
| Tennessee, J. W. Scheibler, D. V. S., 312 Third St., Memphis. | '85 |
| Utah, S. L. Richards, D. V. S., Salt Lake City. | '85 |
| Vermont, S. C. Waterfield, D. V. S., Montpelier | '87 |
| Virginia, J. A. Myers, D. V. S., Linville. | '83 |
| West Virginia, F. B. Ford, D. V. S., Moorefield | '88 |
| Wisconsin, C. Evans, D. V. S., Racine. | '83 |
| Wyoming, A. A. Holcome, D. V. S., Cheyenne | '76 |

FOREIGN CORRESPONDING SECRETARIES.

Cuba, H. T. Laine, D. V. S., Havanna. '86
West Indies, F. W. Peniston, D. V. S., Bermuda. '90
Japan, H. A. Yokura, D. V. S., Koneaba Tokio. '85

A. T. SELLERS, D. V. S., *Secretary.*

